# Editorial: Women in academia: Challenges and solutions to representation in the social sciences

**DOI:** 10.3389/fpsyg.2022.1056350

**Published:** 2022-11-09

**Authors:** Camille S. Johnson, Jessi L. Smith, Colette Van Laar

**Affiliations:** ^1^School of Management, San Jose State University, San Jose, CA, United States; ^2^Office of Academic Affairs and Department of Psychology, University of Colorado Colorado Springs, Colorado Springs, CO, United States; ^3^Department of Psychology, Center for Social and Cultural Psychology, KU Leuven, Leuven, Belgium

**Keywords:** gender equity, psychology, status, intersectionality, discrimination, academia, women—employment, hiring bias

## Introduction

Many scholars and calls to action focus on interventions that address disparities faced by minoritized faculty in the fields of science, technology, engineering, and math (STEM). The fate of women in the social sciences has received much less attention, in part because gender inequities are assumed to not be a problem there. This Research Topic counters these assumptions by providing demonstrations of and examining contributors to gender inequities. First, Fox Tree and Vaid describe the ways in which institutions founded by and for White men do not serve women and racialized faculty and how a focus exclusively on gender prevents understanding intersectional inequities and experiences. In their call for robust datasets (that, for example, go beyond the gender binary) they note that even in fields where there is gender parity, women of color are underrepresented.

Spotlighting demographics is only part of the challenge. Lived experiences also must be considered (and validated). van Veelen and Derks' study of all Dutch universities shows that although women are in the numerical majority in the social and behavioral sciences, women perceive the glass ceiling and estimate lower odds of becoming a full professor compared to their men counterparts. Ollrogge et al., show that in these domains, men and women expect men to be more successful, with men showing hostile sexist attitudes and women experiencing gender-based rejection sensitivity, which mediates personal success expectations. Similarly, Casad et al., summarize the complexities of women's representation and note the leaky pipeline from BA to PhD for women in psychology, anthropology, and sociology, and the consistent predominance of men among economics degree programs at all levels. They reveal gendered patterns of inequity in financial compensation, grant funding, publications, authorship, citations, and speaking roles.

Echoing Fox Tree and Vaid, Morimoto's review of the NSF-ADVANCE program demonstrates how essential intersectionality is (and how it is missing) from most theory-informed interventions for addressing inequities. Morimoto's centering of the importance of NSF's requirement to include an intersectional framework is further emphasized by Wong et al.'s review of women's preferences for interventions and a survey of Dutch organizational diversity interventions. They found that default intervention models focused on white women's needs, but Asian, Black, and Latina women differ in their preferences for interventions that focus on intersectional differences, challenges to authority, and agency.

It is no surprise, then, that interventions in the behavioral and social sciences show mixed results. Research by Täuber highlights how despite years of policies, Dutch women experienced less psychological safety and less positive attitudes toward academic careers. She notes the lack of attention to intersectionality, and the ways in which intersectionality affects inclusion and safety. Rabinowitz and Valian's undergraduate institution case study shows that an infusion of good intentions and funding is insufficient for creating institutional change.

Publishing within the social sciences is also a context in which bias can emerge. As Brown et al. demonstrate, university students afford less value to psychological research published in journals about gender and women, compared to journals on other research topics, with men's lower feminist ideologies predicting lower support for gender journals. In addition, Ashburn-Nardo et al., reveal that the “reproducibility movement” within psychology and other fields has a tremendous impact on faculty careers. Their compelling experimental data shows the overwhelming negative perceptions of social scientists when their research does not replicate and speculate about the consequences on women-identified and faculty of color who are already in vulnerable positions in academia.

## What we have learned

As three social science scholars in different university settings, and parts of the world, we each inhabit a variety of intersectional, although not fully inclusive, identities. In assembling this Research Topic, studies documenting systemic inequities and exploring solutions for women, and women of color in the social sciences, were relatively hard to find. For this Research Topic, scholars intentionally grappled with how their data, even if not specific to social sciences, could inform our understanding of the future of work. The social sciences are integral to understanding and improving the human experience. If people from across the spectrum of gender identities and from different ethnic, racial, and cultural groups are not inclusively engaged in social science scholarship, that scholarship is incomplete and the field unjust.

Importantly, contributing authors worked under extraordinary conditions of gender and racial strife as the publication process unfolded during the global pandemic. To mitigate the stress as much as possible we vowed to be flexible and supportive of our authors and reviewers, who were mostly women, many of whom told us they were caretakers, as they submitted abstracts, manuscripts, and revisions, as well as reviews and comments. In a striking example of how treating people the same does not create equity, we found that editorial systems that remove editorial privilege from the process are built for authors, reviewers, and editors with autonomy and resources. To center the needs of minoritized and marginalized scholars, we extended every single deadline preemptively as well as granted every single extension request. We communicated outside of the publisher's system as much as possible because it sent auto replies and emails that could not be modified. At publication time, we strongly encouraged authors to seek the publication designation that was the least expensive and to request discounts. While open access publication processes allow people to see into the science, which is vital for dissemination and public trust in science, the cost of supporting open access *via* high publications costs limits participation: several potential contributors declined to submit because they lacked the resources to pay the high fees.

Where does this leave us? This Research Topic does more than provide advice for future interventions. The study of gender equity in the social sciences facilitates social scientific discovery, as well as illuminates a specific context. There is more to learn, more assumptions to probe, interventions to design, and publication processes and perceptions to change. We must commit to applying the tools of our sciences to transform our fields. Reading this Research Topic is but one step.

## Author contributions

All authors listed have made a substantial, direct, and intellectual contribution to the work and approved it for publication.

## Conflict of interest

The authors declare that the research was conducted in the absence of any commercial or financial relationships that could be construed as a potential conflict of interest.

## Publisher's note

All claims expressed in this article are solely those of the authors and do not necessarily represent those of their affiliated organizations, or those of the publisher, the editors and the reviewers. Any product that may be evaluated in this article, or claim that may be made by its manufacturer, is not guaranteed or endorsed by the publisher.

